# Evidence of the Disassembly of α-Cyclodextrin-octylamine Inclusion Compounds Conjugated to Gold Nanoparticles via Thermal and Photothermal Effects

**DOI:** 10.3390/molecules21111444

**Published:** 2016-10-29

**Authors:** Nataly Silva, Silvana Moris, Maximiliano Díaz, Nicolás Yutronic, Erika Lang, Boris Chornik, Marcelo J. Kogan, Paul Jara

**Affiliations:** 1 Department of Chemistry, Universidad of Chile, Las Palmeras 3425, Santiago 7800003, Chile; nataly.silva@usach.cl (N.S.); sml2910@ug.uchile.cl (S.M.); mdiazv@ug.uchile.cl (M.D.); nyutroni@uchile.cl (N.Y.); 2 Department of Chemistry of Materials, Universidad de Santiago de Chile, Av. Libertador Bernardo O’Higgins 3363, Santiago 9170022, Chile; 3 Department of Biology, CEM, Universidad of Chile, Las Palmeras 3425, Santiago 7800003, Chile; elang@uchile.cl; 4 Department of Physics, Universidad de Chile, Beauchef 850, Santiago 8370448, Chile; bchornik@ing.uchile.cl; 5 Department of Pharmacological and Toxicological Chemistry, Universidad of Chile, Sergio Livingston 1007, Santiago 8380492, Chile

**Keywords:** inclusion compounds, cyclodextrin, octylamine, gold nanoparticles, photothermal effects

## Abstract

Cyclodextrin (CD) molecules form inclusion compounds (ICs), generating dimers that are capable of encapsulating molecules derived from long-chain hydrocarbons. The aim of this study is to evaluate the structural changes experienced by ICs in solution with increasing temperatures. For this, a nuclear magnetic resonance (^1^H-NMR) titration was performed to determinate the stoichiometric α-cyclodextrin (α-CD):octylamine (OA) 2:1 and binding constant (*k* = 2.16 M^−2^) of ICs. Solution samples of α-CD-OA ICs conjugated with gold nanoparticles (AuNPs) were prepared, and ^1^H-NMR spectra at different temperatures were recorded. Comparatively, ^1^H-NMR spectra of the sample irradiated with a laser with tunable wavelengths, with plasmons of conjugated AuNPs, were recorded. In this work, we present evidence of the disassembly of ICs conjugated with AuNPs. Thermal studies demonstrated that, at 114 °C, there are reversible rearrangements of the host and guests in the ICs in a solid state. Migration movements of the guest molecules from the CD cavity were monitored via temperature-dependent ^1^H-NMR, and were verified comparing the chemical shifts of octylamine dissolved in deuterated dimethylsulfoxide (DMSO-*d*_6_) with the OA molecule included in α-CD dissolved in the same solvent. It was observed that, at 117 °C, OA exited the α-CD cavity. CD IC dimer disassembly was also observed when the sample was irradiated with green laser light.

## 1. Introduction

A variety of guest molecules can be enclosed in the cavities of cyclodextrins (CDs) in order to form inclusion compounds (ICs). The predominant driving forces for the formation of these inclusion compounds are non-covalent hydrophobic effects and van der Waals attractions [1,2,3].

Three possible types of structural packaging have been reporting for α-CD: Cage-type, layer-type, and channel-type. For a channel-type α-CD matrix, two possible arrangements exist for the included guest molecules, namely, head-to-tail and head-to-head orientations [4]. Such CD ICs can form supramolecular assemblies that eventually crystallize in the form of anisotropic polyhedrons, with sizes ranging from the nanometer to the micrometer scale [5].

CD ICs have been extensively studied using different techniques, including differential scanning calorimetry (DSC). This technique corroborates IC formations by comparing the thermal profiles of the isolated precursors with those of the conjugate systems [6,7,8]. However, thermal studies that provide information on CD IC topotactic processes have not been studied.

Presently, CD ICs are widely used in pharmaceutical science and separation technology, and, more recently, they have been adopted in the field of chemical nanostructures [9,10,11]. There are multiple reports focused on the utilization of CD inclusion complexes in their solid state for supporting, and as templates of, metal nanoparticles (MNPs) using long chain alkylthiols, alkylamines, and carboxylic acids as guest molecules. In these compounds, the guest molecules are included in the cyclodextrin, with their functional groups exposed outside of the cavity. This leads to an appropriate surface for the formation and heteroepitaxial growth of MNPs in the crystal (physical method), or for the selective adsorption of MNPs (obtained using chemical methods) [12,13,14,15,16,17].

Novel metal nanoparticles have received particular attention in recent decades for their use in a variety of novel applications. One example is the use of gold nanoparticles (AuNPs), which exhibit great potential as photothermal agents capable of absorbing energy and dissipating said energy within their immediate environment as heat. This property has many potential applications, including in the treatment of cancer, as well as in the selective transport and vectorization of drugs and therapeutic macromolecules acting as “intelligent transport systems”, controlling in space and time, the release of associated therapeutic compounds.

For the in vitro success of cancer therapy, or in the transport and vectorization of drugs using visible light, absorbing nanoparticles can be extended to skin or surface-type cancers [18,19,20]. In this paper, we present thermal studies of cyclodextrin inclusion compounds that account for reversible changes in the system prior to the fusion and decomposition of the supramolecular structure. These changes are attributed to the exit of the guest from the CD cavity, and, possibly, to the disassembly of the IC dimer. The photothermal properties of the AuNPs are employed to transfer energy to the IC for the exiting of the guest molecules from the CD cavity, which implies potential applications in drug-delivery technology.

## 2. Results and Discussion

### 2.1. Inclusion-Compound Preparation, Stoichiometric and Binding Constant Determination

**Figure A1:**
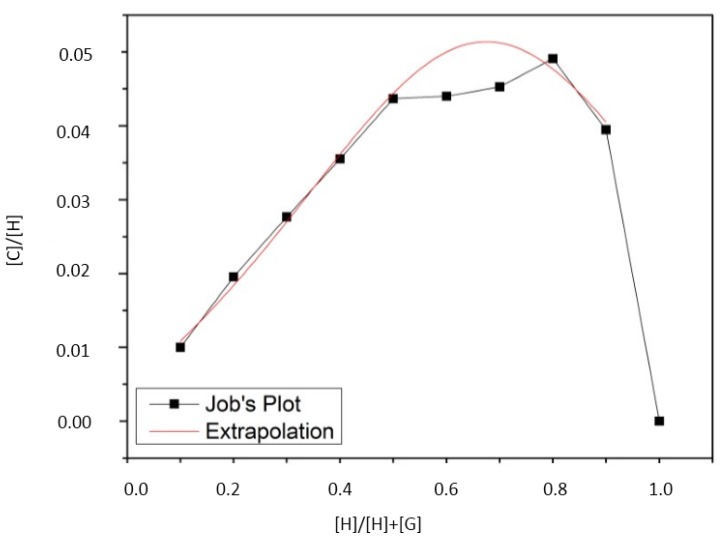
Modified Job’s plot of host–guest complexation using ^1^H-NMR.

^1^H-NMR titration was performed in order to obtain the binding constant of the inclusion complex. A typical Job’s plot procedure [21] was executed in order to obtain the stoichiometry (Figure A1). The peak of complex concentration was found at 0.066 mM of cyclodextrin and 0.033 mM of octylamine, corresponding to a 2:1 stoichiometric ratio. This same data set was used to obtain the binding constant using the modified Rose-Drago procedure, considering the NMR proton shift (Appendix A) [22].

Binding constants were calculated for every titration points used in the Job’s plot, with a wide range of concentration ratios. To obtain the equilibrium constant (Equation (A5)), single-step formation of the complex [H_2_G] was considered, as the ^1^H-NMR chemical shift of the inner host protons and the guest chain protons appear to be too near to each other in 2:1 and 1:1 complexes [23]. The *k* value tends to increase as the [H]/[G] ratio decreases, but has a constant value in the 1.0 ratio; *k* has an average value of 2.16 M^−2^ (Table A1).

### 2.2. UV-Visible Absorption Spectrophotometry

**Figure 1:**
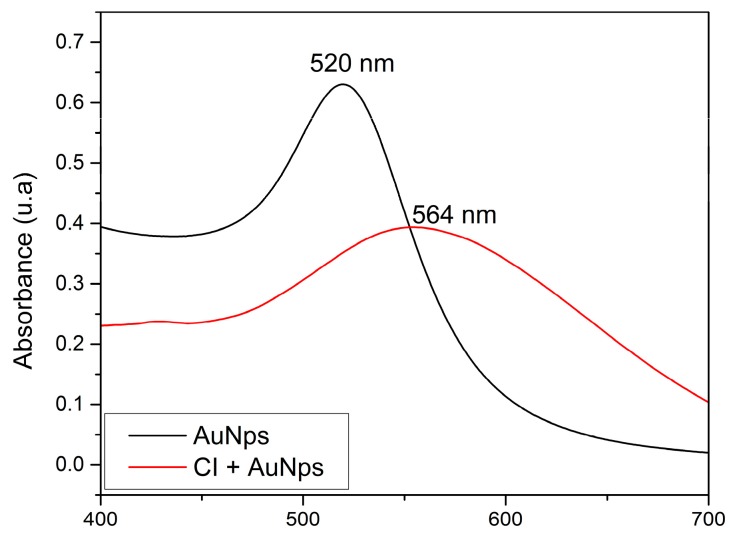
UV-visible absorption spectra of citrate-stabilized AuNPs (recorded in transmission mode), and of AuNPs adhered to the crystal surface of the IC (recorded in diffuse reflectance mode).

Diffuse reflectance spectrophotometry is a useful technique for investigating the adhesion of NPs to the surfaces of organic substrates. Figure 1 presents the absorption spectra of samples of citrate-stabilized colloidal AuNPs and AuNPs, fixed to ICs crystals. For the colloidal AuNPs, an absorption maximum is observed at 520 nm, corresponding to the band that is characteristic of plasmon resonance for spherical gold nanoparticles close to 10 nm in diameter [24]. For AuNPs fixed to ICs crystals, a broadening and a bathochromic shift of this plasmon band is observed at 564 nm. The redshift of the plasmon band can be explained in terms of dipolar coupling between closely neighboring particles, and a change in the dielectric environment. Furthermore, we assume that the formation of AuNPs aggregates onto microcrystals (as observed in scanning electron microscopy (SEM) measurements) is consistent with the broadening of the absorption band [24].

### 2.3. Transmission Electronic Microscopy (TEM)

**Figure 2:**
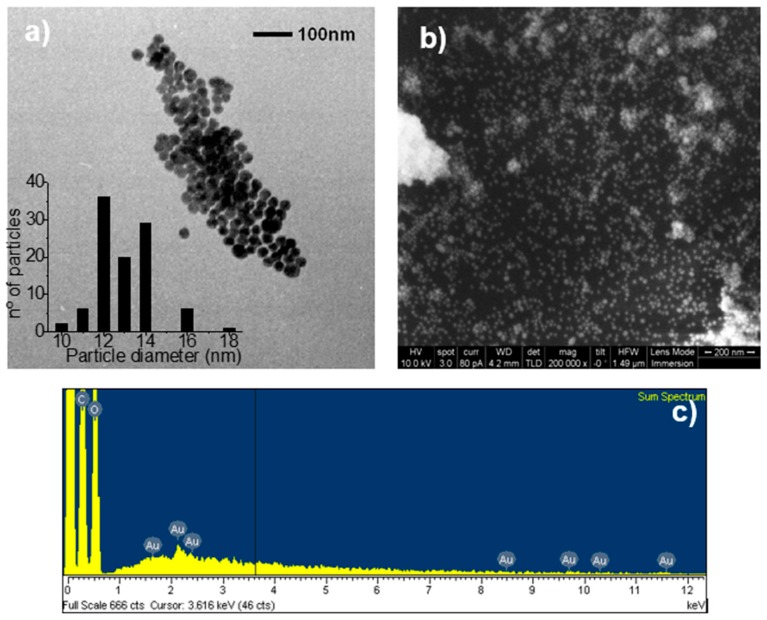
(**a**) TEM micrograph and histogram; (**b**) SEM micrograph; and (**c**) energy-dispersive X-ray spectroscopy (EDS) spectrum of α-CD/OA IC conjugated to AuNPs.

Figure 2a presents a TEM micrograph and the corresponding size-distribution histogram of AuNPs that are adhered to the crystal surface of the IC. Homogeneous distributions in size and shape are observed. The histogram, which was obtained from a population of 100 particles, indicates an average diameter of 12 ± 1.3 nm. These results indicate that the particles, which were synthesized using the Turkevich method, retained their original sizes and morphologies (Figure S1).

### 2.4. Scanning Electronic Microscopy (SEM) and Energy-Dispersive X-ray Spectroscopy (EDS)

Figure 2b presents an SEM micrograph and the EDS analysis of AuNPs adhered to IC crystals. A large population of particles of homogeneous size and shape distributions, attached to the IC, is observed. Aggregates of NPs are also observed in some areas, which is consistent with the results obtained through diffuse reflectance spectrophotometry. EDS analysis indicates the presence of C and O in the IC. The presence of gold confirms the adhesion of the metal particles.

### 2.5. X-ray Photoelectron Spectroscopy (XPS)

In the study of the selective adhesion of AuNPs to organic crystals, it is very important to determine the type of interaction between the functional group of the guest molecule (which functions as a surfactant) and the surface of the metal NP. XPS spectroscopy is employed for this purpose.

**Figure 3:**
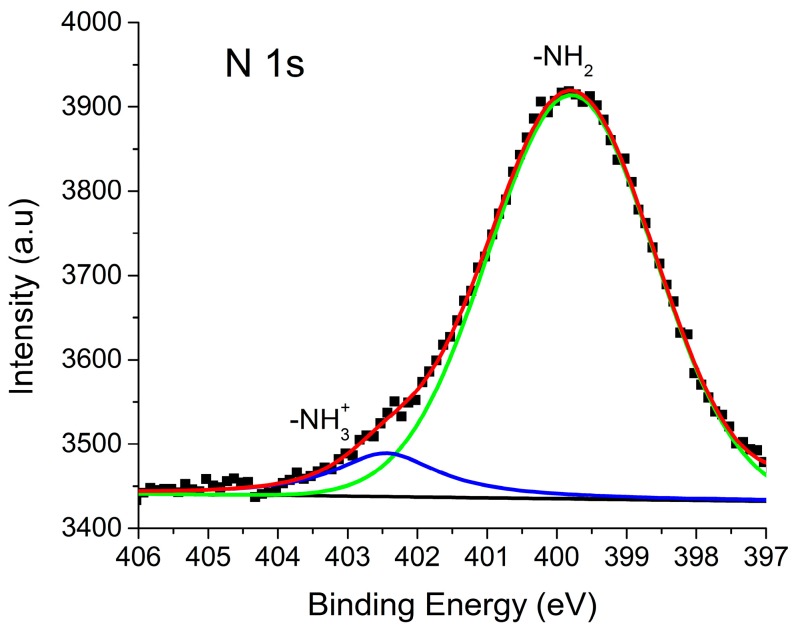
High-resolution XPS spectra with peaks that correspond to the binding energies of nitrogen 1s electrons of OA in a sample of α-CD IC crystals with adhered AuNPs. The original spectrum (black dots lined with red) and the fitted curves (green line assigned to the -NH_3_^+^ group and blue line assigned to the -NH_2_ group) are shown. The XPS general spectrum is shown in the Supplementary Materials.

Figure 3 presents a high-resolution XPS spectrum of the nitrogen 1s band of OA in a sample of α-CD IC crystals with adhered AuNPs (Figure S3 shows XPS general spectrum). Curve fitting provides two well-defined peaks at 399.9 and 402.4 eV. The peak with a lesser intensity, at 402.4 eV, is assigned to the nitrogen atom of the ammonium ion (-NH_3_^+^) of the OA molecule (which is protonated, consistent with a pKa of 10.75 at pH 7). The peak with a higher intensity and lower binding energy, at 399.9 eV, is assigned to the -NH_2_ groups that are adsorbed on the AuNPs, thereby increasing the electron density of the nitrogen atom [25,26,27,28]. Pong et al. calculated the energy associated with the nitrogen interaction with different planes and ridges for the AuNPs, obtaining energies between 3.4 and 9.2 kcal/mol. These binding energies are associated with weak interactions of ion-induced dipole types, with the ammonium ion and AuNPs acting as a dipole [29,30].

### 2.6. Thermal Analysis

**Figure 4:**
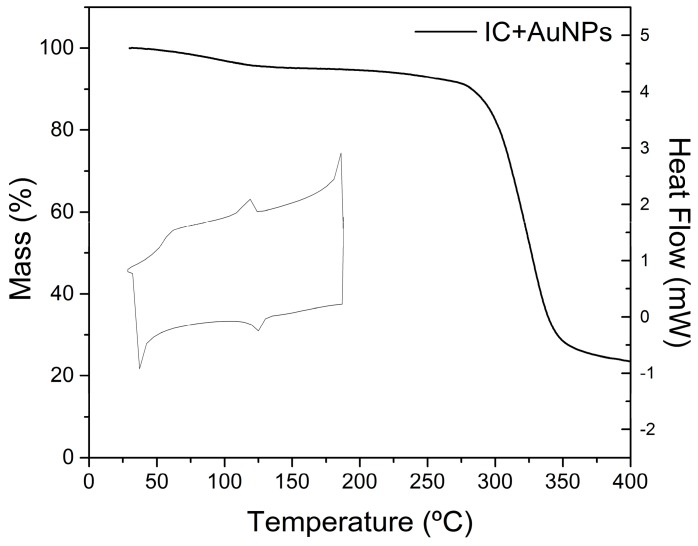
Thermogram (DSC/TGA) of the α-CD/OA IC crystals with fixed AuNPs. The temperature range was between 25 and 400 °C for TGA, and between 25 and 200 °C for DSC, with a heating rate of 10 °C·min^−1^.

Figure 4 presents the thermogram (DSC/TGA) of α-CD IC crystals with adhered AuNPs. An endothermic peak at 124.3 °C and an exothermic peak at 131 °C are observed. This result differs from that obtained from the IC without AuNPs, where two endothermic peaks and two exothermic peaks were observed. This is due to fact that the presence of the particles provides some rigidity to the IC (Figure S2). Analysis of the TGA data indicates an absence of mass loss associated with these thermal events. A mass loss of 4.31% is observed between 25 and 113.2 °C, and is attributed to the loss of moisture water molecules in the IC.

The endothermic and exothermic peaks are attributed to conformational, reversible changes of the host–guest system. These are most likely associated with migration movements of the guest molecules into the cyclodextrin cavity, where the integrity of the structure is maintained.

### 2.7. Variable-Temperature ^1^H-NMR Study

Possible migration movements of the guest molecules into the cyclodextrin cavity were monitored through a ^1^H-NMR variable-temperature study. For this, a solid-state sample of nanoparticles, adhered to the inclusion compound crystal, were dissolved in DMSO-*d*_6_. This high polarity solvent has a weak affinity to the cyclodextrin cavity compared to the octylamine guest, the complex structure remaining unaltered. Furthermore, DMSO-*d*_6_ not interchange with the CD hydroxyl groups. When a sample is dissolved, an IC rearrangement occurs and the AuNPs are completely surrounded by the IC, interacting through the NH_2_ of the guest molecules. Thus, a NP measuring 12 nm in diameter can remain in a DMSO-*d*_6_ solution, being surrounded by a maximum of 368 cyclodextrin units, and therefore, an equal number of OA (i.e., the conjugation of CD to NPs). This number was obtained by dividing the surface area of a spherical particle with a 12-nm diameter by the area of the base of the cyclodextrin unit (outer diameter, 1.46 nm [1]).

**Figure 5:**
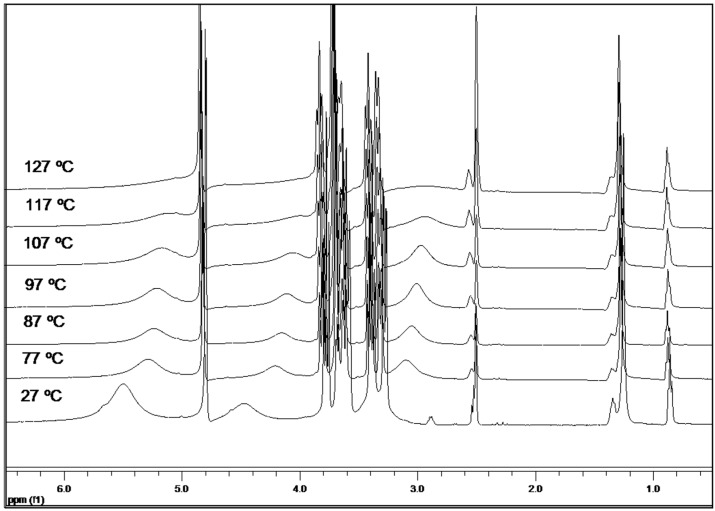
^1^H-NMR spectra of a α-cyclodextrin/octylamine IC conjugated with AuNPs at various temperatures.

**Figure 6:**
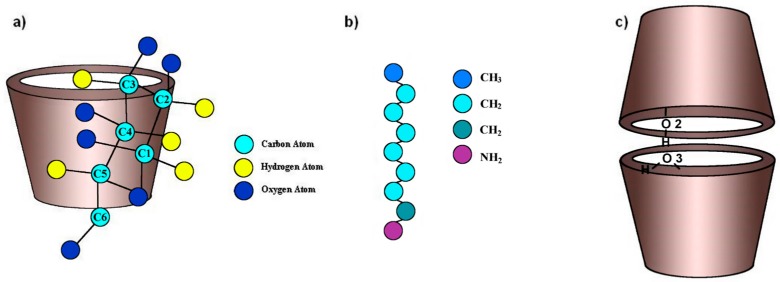
Schematic representations of: (**a**) α-CD; (**b**) octylamine guest molecule; and (**c**) inclusion-compound dimer.

Figure 5 shows that the host and guest protons experience chemical shifts when the temperature is increased. Table 1 summarizes the observed chemical shifts of the α-CD protons. The protons, H4, H3, H5, and H6, are shifted to lower fields with increasing temperatures, because of the lower electron density present in the CD cavity after the exit of the guest molecules. Those that are outside of the cavity, OH6, H1, OH2, and OH3, are shifted upfield. This shift is caused by the disruption of the hydrogen bonds between adjacent cyclodextrin units (Figure 6).

Furthermore, it is observed that all protons of the OA guest molecules experience chemical shifts to lower fields with increasing temperatures (Table 2). The chemical shifts of the OA molecules in the IC at 117 °C are equivalent to the chemical shifts of a free OA molecule dissolved in DMSO-*d*_6_, which provides evidence of the exit of this guest molecule from the matrix cavity of CD. All hydrogen atoms of the OA molecule have sharp signals without splitting up to 117 °C. This leads to the conclusion that all guest molecules have been released from the cavity of the cyclodextrin at this temperature.

Temperature-dependent ^1^H-NMR experiments performed on ICs without AuNPs, yielded the same results. Therefore, the incorporation of AuNPs does not interfere in the conformational changes undergone by the IC in solution with increasing temperatures. However, the signals for CD IC protons are broadened. This type of signal broadening has been previously reported in alkanethiolate-capped gold clusters [31,32]. A fast relaxation and environmental heterogeneities are thought to be, potentially, responsible for these line broadening effects (Figure S4).

CD molecules have a high capacity for binding water molecules via hydrogen bonds to the hydroxyl groups at the edges of the structure, and also for undergoing anisotropic movements. The signal that appears with increasing temperature at approximately 3.1 ppm is assigned to the hydrogen atoms of the water molecules present in the CD. The absence of this signal at room temperature indicates that the water molecules are not from the solvent. An increase in temperature causes the release of these water molecules due to the rupturing of the hydrogen bonds, thereby increasing electron density around the hydrogen atom, and shifting the proton signal to higher fields decreases its intensity (most likely due to the evaporation of the water). This signal is not observed at 27 °C because of the overlap with the signals of the hydrogen atoms of the CD.

After the heating process, the sample was cooled to 27 °C, and a new ^1^H-NMR spectrum was recorded. All chemical shifts, both of the host and guest molecules, were found to be equivalent to the chemical shifts of the IC before the heating process (Figure S5 and Tables S1 and S2). These results are consistent with a reversible process in which the guest molecules exit the matrix cavity as the temperature increases.

This result confirms that the reversible phase changes observed in studies based on differential scanning calorimetry are attributable to the exit of the guest octylamine from the cavity of the α-CD matrix at elevated temperatures.

**Figure 7:**
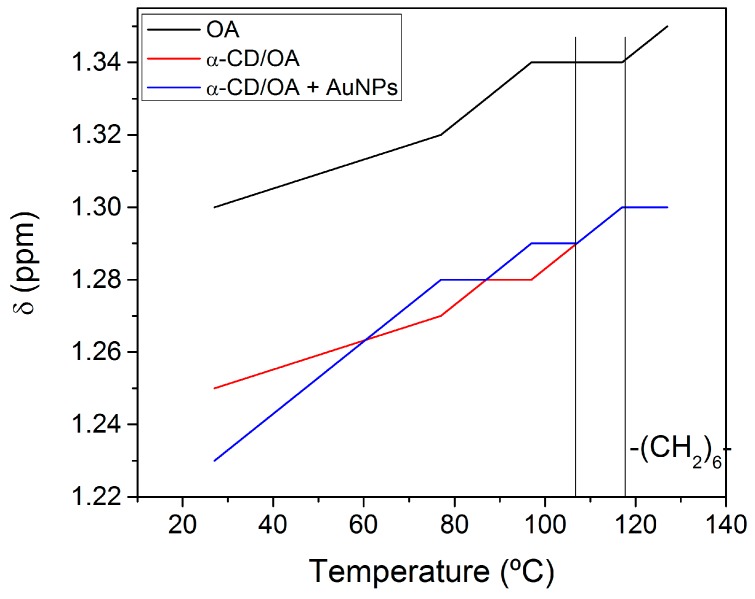
Graph of chemical shifts of (CH_2_)_6_ groups versus temperature. The black, red, and blue lines correspond to OA dissolved in DMSO-*d*_6_, α-CD/OA IC, and α-CD/OA IC conjugated with AuNPs, respectively.

Figure 7 presents a graph of the chemical shifts of the (CH_2_)_6_ groups of OA dissolved in DMSO-*d*_6_, OA included in α-CD, and α-CD/OA IC conjugated with AuNPs as a function of temperature.

In all cases, an inflection point at 77 °C is observed, which is attributed to a conformational change (most likely stretching) of the alkyl chain of the OA.

Analysis of the region between 107 °C and 117 °C reveals downfield shifts for the protons of OA included within CD (with and without NPs). These shifts are attributed to the release of the guest molecules from the matrix cavity. The free OA molecule (dissolved in DMSO-*d*_6_) remains unchanged because it is in an isotropic medium. This experiment shows that the conjugation of the particle to the IC does not interfere with the conformational change, which does not affect the release of the guest molecule due to the temperature effect.

### 2.8. ^1^H-NMR Study of Photothermal Effects

**Figure 8:**
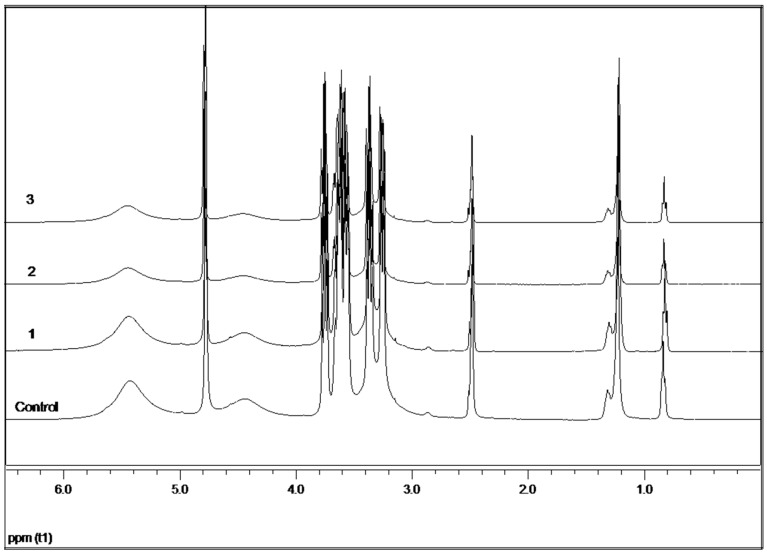
^1^H-NMR spectra of IC conjugated with AuNPs, before and after irradiation. The irradiation conditions for each spectra were: Control (without irradiation), 1 (250 mW/15 min), 2 (450 mW/15 min), and 3 (450 mW/60 min).

Figure 8 presents the ^1^H-NMR spectra of a sample of conjugated α-CD/OA/AuNPs, before and after irradiation, for varying exposure times and laser powers. It is observed that the signals of the OH2 and OH3 groups undergo a broadening and an intensity decrease, because of the weakening of the hydrogen bonds between the -OH groups of the assembled cyclodextrins. Additionally, the OH6 undergoes a broadening and an intensity decrease due to the distancing of the AuNPs. This variation in the signal is generated by the anisotropic local heating produced by laser-irradiated AuNPs, which mainly affects dimers that are conjugated directly with the AuNPs, resulting only in a broadening of the signals from the OH groups. This effect does not cause changes in the rest of the guest protons or the inner protons of the CD cavity due to the low temperatures reached. ^1^H-NMR experiments performed with IC, without AuNPs, show that the radiation does not affect spectral signals, confirming that local heating is exclusively due to the presence of gold particles (Figure S6).

## 3. Materials and Methods

### 3.1. Inclusion-Compound Preparation, Stoichiometric and Binding Constant Determination

All chemical reagents are commercially available and were used as received: Octylamine (Sigma-Aldrich, Steinheim, Germany), α-cyclodextrin (Sigma-Aldrich, Saint Louis, MO, USA), HAuCl_4_ (Sigma-Aldrich), Na_3_C_6_H_5_O_7_ (Sigma-Aldrich), and nanopure water (Merck, Darmstadt, Germany).

The IC crystals were prepared following previously described protocols [33,34]. The IC was obtained by directly mixing OA (255 μL) with an α-CD solution (1.0 g/10 mL, water). The alkylamine was added dropwise under constant stirring. Precipitation of a fine white powder was indicative of IC formation. After two days, the crystalline powder was filtered and washed with nanopure water in order to remove any traces of uncomplexed α-CD. The crystals were subsequently washed with 15 mL of methanol to remove excess OA. Finally, the compounds were dried under vacuum for four hours (Yields: α-CD/OA, 76.3%).

*Synthesis of colloidal gold nanoparticles.* The Au colloid was synthesized in accordance with the Turkevich method [35]. In a 250-mL round-bottomed flask equipped with a condenser, 100 mL of an aqueous HAuCl_4_ solution (1 mM) was brought to boil, under vigorous stirring. As quickly as possible, 10 mL of Na_3_C_6_H_5_O_7_ solution (38.8 mM) was added to the solution under continuous stirring. The solution was heated for an additional 30 min and then allowed to cool to room temperature. Then, the solution was filtered through a 0.45-μm cellulose acetate membrane filter, yielding 12-nm diameter AuNPs.

*Conjugation of IC to AuNPs*. The sample of IC crystals (0.1 g) was immersed in a solution of AuNPs (500 μL) at room temperature (at pH 7) and was subsequently washed with a 38.8 nM (500 μL) citrate solution to remove AuNPs that had not adhered. Finally, the sample of AuNPs that had adhered to the IC crystals was dried under vacuum [24].

### 3.2. UV-Visible Absorption Spectroscopy

A Shimadzu UV-3101PC spectrophotometer (Shimadzu, Kyoto, Japan) was used. Spectra were recorded between 400 and 700 nm. The diffuse reflectance results were converted into absorbance units using Kubelka-Munk conversion.

### 3.3. Transmission Electronic Microscopy

TEM was performed using a Zeiss model EM-109 microscope (Jena, Germany) operating at 50 kV.

### 3.4. Scanning Electronic Microscopy and Energy-Dispersive X-ray Spectrometry

SEM images were obtained using a high-resolution LEO 1420VP instrument (Zeiss, Jena, Germany) equipped with an energy-dispersive detector.

### 3.5. X-ray Photoelectron Spectroscopy

XPS spectra were recorded using a photoelectron spectrometer model 1257 (Perkin Elmer, Physical Electronic Division, Eden Prairie, MN, USA), fitted with an ultra-high-vacuum main chamber, a hemispherical electron analyzer, and an X-ray source that provided unfiltered Al Kα radiation. Energy calibration was accomplished by assigning a binding energy of 284.8 eV to the C 1s peak of adventitious carbon.

### 3.6. Thermal Analysis

Thermogravimetric Analysis (TGA): Analysis was performed using a thermobalance TGA-SDTA 851e/SF/1100 (Mettler Toledo, Greifensee, Switzerland). The sample was placed in an aluminum capsule. Nitrogen was used as the purge gas, and the temperature range was between 25 and 400 °C, with a heating rate of 10 °C·min^−1^.

Differential Scanning Calorimetry (DSC): Scans were performed using a DSC-822e/400 (Mettler Toledo, Greifensee, Switzerland), with an aluminum capsule sample used as the holder and reference. Nitrogen was used as the purge gas, and the temperature range cycled between 25 °C and 200 °C, with a heating rate of 10 °C·min^−1^.

### 3.7. ^1^H Nuclear Magnetic Resonance (^1^H-NMR)

The spectra were obtained in a Bruker Advance 400 MHz superconducting NMR spectrometer (Rheinstetten, Germany) using DMSO-*d*_6_ as solvent and internal reference. Twenty-five milligrams of sample were dissolved in 0.795 mL of DMSO-*d*_6_.

### 3.8. ^1^H-NMR Study of Photothermal Effects

In the irradiation study, a sample of α-CD IC crystals with adhered AuNPs (26.7 mg) was dissolved in 0.795 mL of DMSO-*d*_6_. The solution was maintained under constant stirring in a ^1^H-NMR tube, and was irradiated with a continuous wave laser (532 nm wavelength). The irradiation conditions for each spectrum were: Control (without irradiation), 1 (250 mW/15 min), 2 (450 mW/15 min), and 3 (450 mW/60 min).

## 4. Conclusions

DSC analysis reveals a reversible phase change of α-CD IC crystals with adhered AuNPs. Because this phase change is not accompanied by a loss of mass, it is attributable to a structural change in the IC. This conclusion was verified through a variable-temperature ^1^H-NMR study.

The protons of the guest molecules and the protons of the α-CD that are within the cavity of the IC both experienced a downfield chemical shift. These shifts are evidence of reversible structural modifications. The shifts are caused by the loss of electron density, which results from the migration of the guest molecules out of the matrix cavity at elevated temperature. This phenomenon is, therefore, a topotactic transition. α-CD IC dimer disassembly was also observed when the sample was irradiated with a green laser light. These phenomena have potential applications in drug delivery.

## Figures and Tables

**Table 1:** Chemical shifts of α-CD in an IC conjugated with AuNPs at various temperatures.

Temperature	H1	H2	H3	H4	H5	H6	OH(2)	OH(4)	OH(6)
(°C)	(ppm)	(ppm)	(ppm)	(ppm)	(ppm)	(ppm)	(ppm)	(ppm)	(ppm)
27	4.79	3.27	3.77	3.38	3.59	3.65	5.49	5.49	4.47
77	4.82	3.31	3.81	3.40	3.62	3.61	5.28	5.28	4.20
87	4.82	3.32	3.81	3.41	3.63	3.70	5.24	5.24	4.15
97	4.83	3.34	3.82	3.41	3.64	3.70	5.20	5.20	4.11
107	4.83	3.33	3.82	3.41	3.64	3.71	5.15	5.15	4.06
117	4.84	3.34	3.83	3.42	3.65	3.72	5.05	5.05	4.01
127	4.84	3.35	3.83	3.42	3.66	3.73			

**Table 2:** Chemical shifts of pure OA and OA included in α-CD at various temperatures.

Sample	Temperature (°C)	CH_3_ (ppm)	-(CH_2_)*_n_*- (ppm)	-CH_2_- (ppm)	NH_2_ (ppm)
α-CD/OA	27	0.85	1.23	1.31	2.53
	77	0.89	1.28	1.36	2.54
	87	0.88	1.28	1.36	2.55
	97	0.88	1.29	1.36	2.55
	107	0.89	1.29	1.37	2.56
	117	0.89	1.30	1.37	2.56
	127	0.89	1.30	1.37	2.57
OA	27	0.89	1.30	1.37	2.56

## Supplementary Materials

Supplementary materials can be accessed at: http://www.mdpi.com/1420-3049/21/11/1444/s1.

## Appendix A

Job’s plot was executed using NMR titration using the standard procedure, plotting [C]/[H] vs. [H]/[H] + [G], where [C], by the chemical shift of the H5 inner proton between the free and complexed cyclodextrin (vide infra), was obtained. A notable depression is observed in the high segment of the plot. An extrapolation was realized considering that the 2:1 complex does not present a significant difference in the chemical shift compared to a 1:1 complex.

For the binding constant calculation, a typical 2:1, one-step formation was considered:

*G + 2H* ↔ *H*_2_*G*

(A1) $$k=\frac{\left[H_{2}G\right]}{{\left[H\right]}^{2}*\;\left[G\right]}$$

in equilibrium:

(A2) $$k=\frac{\left[C\right]}{{\left[H_{t}-2C\right]}^{2}*\;\left[G_{t}-C\right]}$$

where *H* is the host; *G* is the guest; *C* is the complex; *H_t_* and *G_t_* are the known concentrations before equilibrium, and the complex concentration is obtained by comparing the chemical shift of the inner protons in the free and complexed cyclodextrin. Because there is no observable difference in the H5 proton shifts for the 1:1 and 2:1 complexes, is not possible to determine the stepwise 1:1 and 2:1 complexation constants. In this case, the host–guest complexation equilibrium has a fast exchange rate in comparison to the NMR time scale; therefore, it is not possible to observe independent peaks for 1:1 and 1:2 complexes and free cyclodextrin. Instead, just a single signal is observed, of which the chemical shift is in direct relation with the molar ratio between the free and CD complexes.

(A3) $$\delta =\delta_{c}\cdot \left[C\right]+\delta_{H}\cdot \left[H_{t}-2C\right]$$

(A4) $$\left[C\right]=\frac{\delta_{n}-\delta_{H}}{\delta_{C}-\delta_{H}}\cdot \frac{{\left[H\right]}_{t}}{2}$$

where δ*_n_* is the observed shift, δ*_H_* is the free cyclodextrin shift, and δ*_c_* is the complex shift. From Equations (A2) and (A3), the following equation is derived:

(A5) $$\frac{1}{k}=-{S_{n}}^{2}{{\left[H\right]}_{t}}^{2}+4\left[H\right]\left(\left[H\right]+\left[G\right]\right)\left(\frac{S_{n}}{2}-1\right)+\frac{2\left[H\right]\left[G\right]}{S_{n}}$$

where

(A6) $$S_{n}=\frac{\delta_{n}-\delta_{H}}{\delta_{C}-\delta_{H}}$$

using:

**Table A1:** Binding constant (*k*) calculated for every concentration used in the plot.

[H] M	[G] M	[H]/[G]	*k*·M^−2^
0.01	0.09	0.11	8.90
0.02	0.08	0.25	4.56
0.03	0.07	0.43	3.23
0.04	0.06	0.67	2.53
0.05	0.05	1.00	2.06
0.06	0.04	1.50	2.06
0.07	0.03	2.33	2.01
0.08	0.02	4.00	1.87
0.09	0.01	9.00	2.38

## References

[1] Sá Couto A., Salústio P., Cabral-Marques H., Ramawat G.K., Mérillon J.M. (2015). Polysaccharides: Bioactivity and Biotechnology.

[2] D’Souza V., Lipkowitz K. (1998). Cyclodextrins. Chem. Rev..

[3] Cabral Marques H.M. (2010). A review on cyclodextrin encapsulation of essential oils and volatiles. Flavour Fragr. J..

[4] Rodríquez-Llamazares S., Yutronic N., Jara P., Englert U., Noyong M., Simon U. (2007). The Structure of the First Supramolecular a-Cyclodextrin Complex with an Aliphatic Monofunctional Carboxylic Acid. Eur. J. Org. Chem..

[5] Herrera B., Adura C., Yutronic N., Kogan M., Jara P. (2013). Selective nanodecoration of modified cyclodextrin crystals with gold nanorods. J. Colloid Interface Sci..

[6] Grandelli H.E., Stickle B., Whitetington A., Kiran E. (2013). Inclusion complex formation of β-cyclodextrin and Naproxen: A study on exothermic complex formation by differential scanning calorimetry. J. Incl. Phenom. Macrocycl. Chem..

[7] Lauro M.R., Carbone C., Auditore R., Musumeci T., Santagati N.A., Aquino R.P., Puglisi G. (2013). A new inclusion complex of amlodipine besylate and soluble β-cyclodextrin polymer: Preparation, characterization and dissolution profile. J. Incl. Phenom. Macrocycl. Chem..

[8] Liu B., Li W., Zhao J., Liu Y., Zhu X., Liang G. (2013). Physicochemical characterization of the supramolecular structure of luteolin/ cyclodextrin inclusion complex. Food Chem..

[9] Martin Del Valle E.M. (2004). Cyclodextrins and their uses: A review. Process Biochem..

[10] Loftsson T., Duchêne D. (2007). Cyclodextrins and their pharmaceutical applications. Int. J. Pharm..

[11] Aiassa V., Zoppi A., Albesa I., Longhi M.R. (2015). Inclusion complexes of chloramphenicol with B-cyclodextrin and aminoacids as a way to increase drug solubility and modulate ROS production. Carbohydr. Polym..

[12] Liu Y., Male K.B., Bouvrette P., Luong J.H.T. (2003). Control of the size and distribution of gold nanoparticles by unmodified cyclodextrins. Chem. Mater..

[13] Barrientos L., Yutronic N., Del Monte F., Gutiérrez M.C., Jara P. (2007). Ordered arrangement of gold nanoparticles on an α-cyclodextrin-dodecanethiol inclusion compound produced by magnetron sputtering. New J. Chem..

[14] Silva N., Moris S., Herrera B., Díaz M., Kogan M., Barrientos L., Yutronic N., Jara P. (2010). Formation of Copper Nanoparticles Supported onto Inclusion Compounds of a-cyclodextrin: A New Route to Obtain Copper Nanoparticles. Mol. Cryst. Liq. Cryst..

[15] Díaz M., Silva N., Yutronic N., Peña E., Chornik B., Jara P. (2014). γ-Cyclodextrin/alkylthiol inclusion compounds crystals as substrates for the formation and immobilization of gold nanoparticles produced by magnetron sputtering. J. Incl. Phenom. Macrocycl. Chem..

[16] Silva N., Arellano E., Castro C., Yutronic N., Lang E., Chornik B., Jara P. (2015). Cyclodextrin inclusion compound crystals for growth of Cu-Au core-shell nanoparticles. J. Incl. Phenom. Macrocycl. Chem..

[17] Silva N., Muñoz C., Diaz-Marcos J., Samitier J., Yutronic N., Kogan M.J., Jara P. (2016). In Situ Visualization of the Local Photothermal Effect Produced on α-Cyclodextrin Inclusion Compound Associated with Gold Nanoparticles. Nanoscale Res. Lett..

[18] Skirtach A.G., Dejugnat C., Braun D., Susha A.S., Rogach A.L., Parak W.J., Möhwald J., Sukhorukov G.B. (2005). The Role of Metal Nanoparticles in Remote Release of Encapsulated Materials. Nano Lett..

[19] Pissuwan D., Valenzuela S.M., Cortie M.B. (2006). Therapeutic possibilities of plasmonically heated gold nanoparticles. Trends Biotechnol..

[20] Jain P.K., El-Sayed I.H., El-Sayed M.A. (2007). Au nanoparticles target cancer. Nano Today.

[21] Hirose K. (2001). A Practical Guide for the Determination of Binding Constants. J. Incl. Phenom. Macrocycl. Chem..

[22] Simova S., Berger S. (2005). Diffusion measurements vs. chemical shift titration for determination of association constants on the example of camphor-cyclodextrin complexes. J. Incl. Phenom..

[23] Dodziuk H., Nowinski K.S., Kozminski W., Dolgonos G. (2003). On the impossibility of determination of stepwise binding constants for the 1:2 complex of (+)-camphor with α-cyclodextrin. Org. Biomol. Chem..

[24] Rodríguez-Llamazares S., Yutronic N., Jara P., Noyong M., Bretschneider J., Simon U. (2007). Face Preferred Deposition of Gold Nanoparticles on α-Cyclodextrin/Octanethiol Inclusion Compound. J. Colloid Interface Sci..

[25] Wojciech Fabianowski S.L.R., Coyle L.C., Weber B.A., Granata R.D., Castner D.G., Sadownik A. (1989). Spontaneous assembly of phosphatidylcholine monolayers via chemisorption onto gold. Langmuir.

[26] Petrovykh D.Y., Kimura-Suda H., Whitman L.J., Tarlov M.J. (2003). Quantitative analysis and characterization of DNA immobilized on gold. J. Am. Chem. Soc..

[27] Techane S.D., Gamble L.J., Castner D.G. (2011). X-ray photoelectron spectroscopy characterization of gold nanoparticles functionalized with amine-terminated alkanethiols. Biointerphases.

[28] Baio J.E., Weidner T., Brison J., Graham D.J., Gamble L.J., Castner D.G. (2009). Amine Terminated SAMs: Investigating Why Oxygen is Present in these Films. J. Electron Spectrosc. Relat. Phenom..

[29] Pong B.K., Lee J.Y., Trout B.L. (2005). First principles computational study for understanding the interactions between ssdna and gold nanoparticles: Adsorption of methylamine on gold nanoparticulate surfaces. Langmuir.

[30] Atkins P., de Paula J. (2009). Physical Chemistry.

[31] Liu J., Alvarez J., Ong W., Román E., Kaifer A.E. (2001). Phase transfer of hydrophilic, cyclodextrin-modified gold nanoparticles to chloroform solutions. J. Am. Chem. Soc..

[32] Hostetler M.J., Wingate J.E., Zhong C.J., Harris J.E., Vachet R.W., Clark M.R., Londono J.D., Green S.J., Stokes J.J., Wignall G.D. (1998). Alkanethiolate gold cluster molecules with core diameters from 1.5 to 5.2 nm: core and monolayer properties as a function of core size. Langmuir.

[33] Barrientos L., Lang E., Zapata-Torres G., Celis-Barros C., Orellana C., Jara P., Yutronic N. (2013). Structural elucidation of supramolecular alpha-cyclodextrin dimer/aliphatic monofunctional molecules complexes. J. Mol. Model..

[34] Barrientos L., Allende P., Orellana C., Jara P. (2012). Ordered arrangements of metal nanoparticles on alpha-cyclodextrin inclusion complexes by magnetron sputtering. Inorg. Chim. Acta.

[35] Turkevich J., Stevenson P.C., Hillier J. (1951). The size and shape factor in colloidal systems. Discuss. Faraday Soc..

