# Supplementary material for: Evidence of the Disassembly of α-Cyclodextrin-octylamine Inclusion Compounds Conjugated to Gold Nanoparticles via Thermal and Photothermal Effects
Source: Molecules. 2016 Oct 29;21(11):1444. doi: 10.3390/molecules21111444 (PMC6273894; doi:10.3390/molecules21111444)
Supplement: Supplementary file 1 [file molecules-21-01444-s001.pdf]

Supplementary Materials: Evidence of the Disassembly of α-Cyclodextrin-octylamine Inclusion Compounds Conjugated to Gold Nanoparticles via Thermal and Photothermal Effects

Nataly Silva, Silvana Moris, Maximiliano Díaz, Nicolás Yutronic, Erika Lang, Boris Chornik, Marcelo J. Kogan and Paul Jara

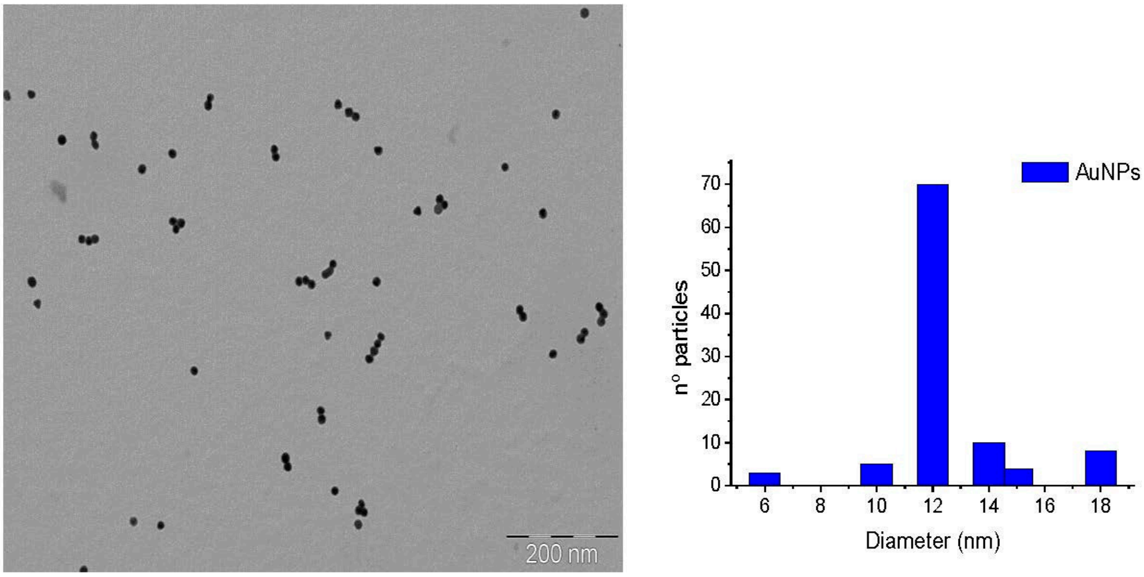

Figure S1. TEM micrograph and histogram of gold nanoparticles.

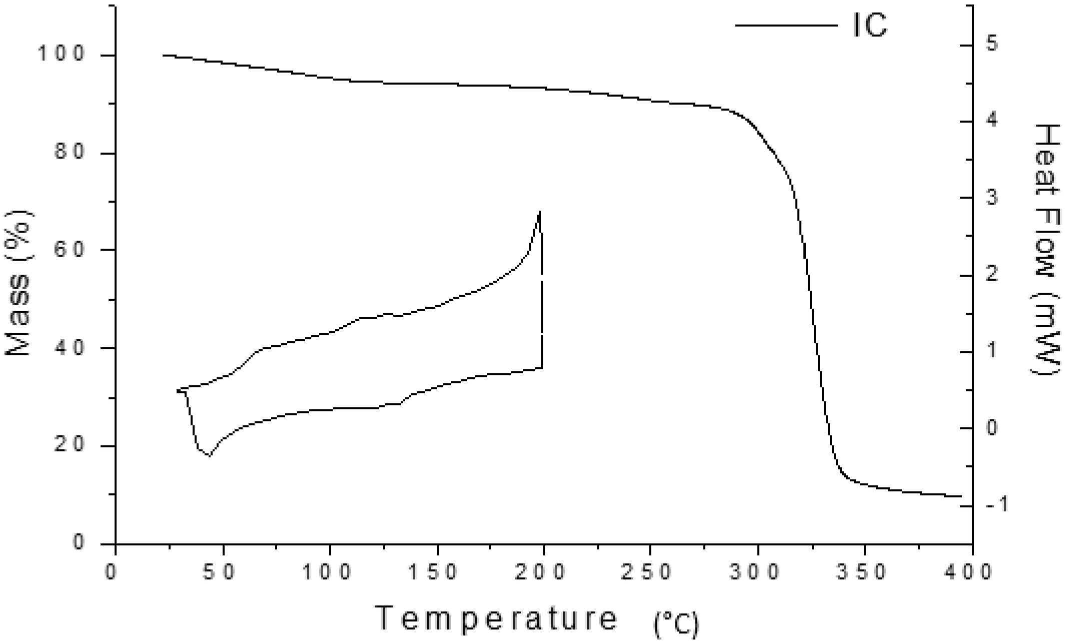

Figure S2. Thermogram (DSC/TGA) of the α-cyclodextrin/octylamine inclusion compound. The temperature range was between 25 and 400 °C for TGA and between 25 and 200 °C for DSC, with a heating rate of 10 °C··min^{-1}.

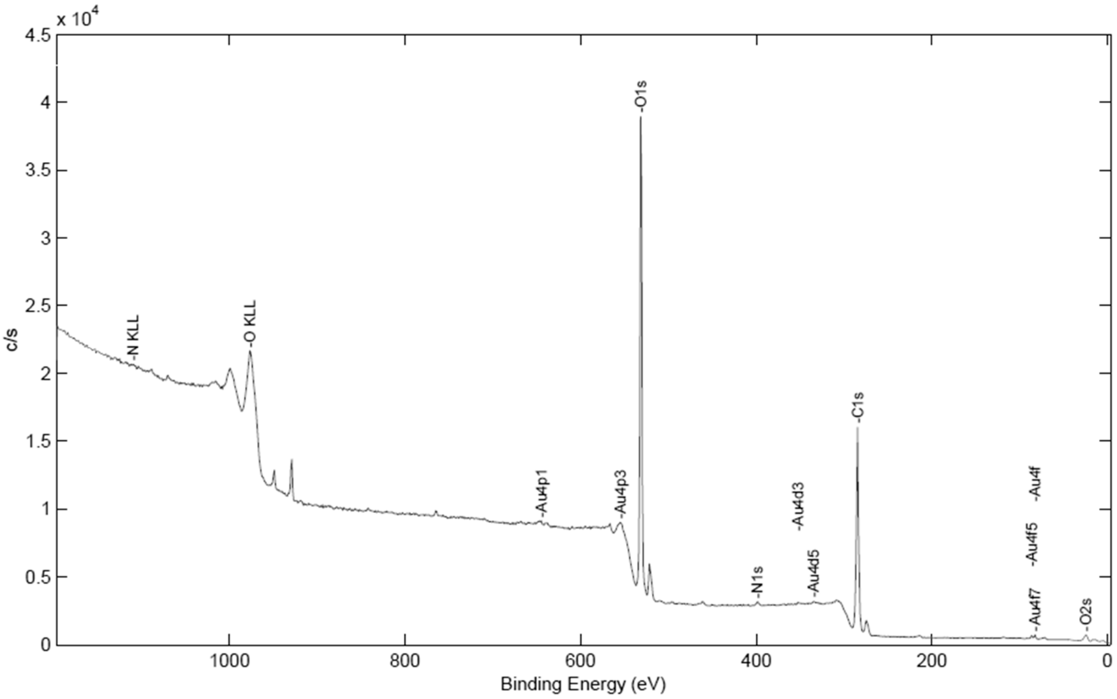

Figure S3. XPS general spectrum of IC conjugated to AuNPs.

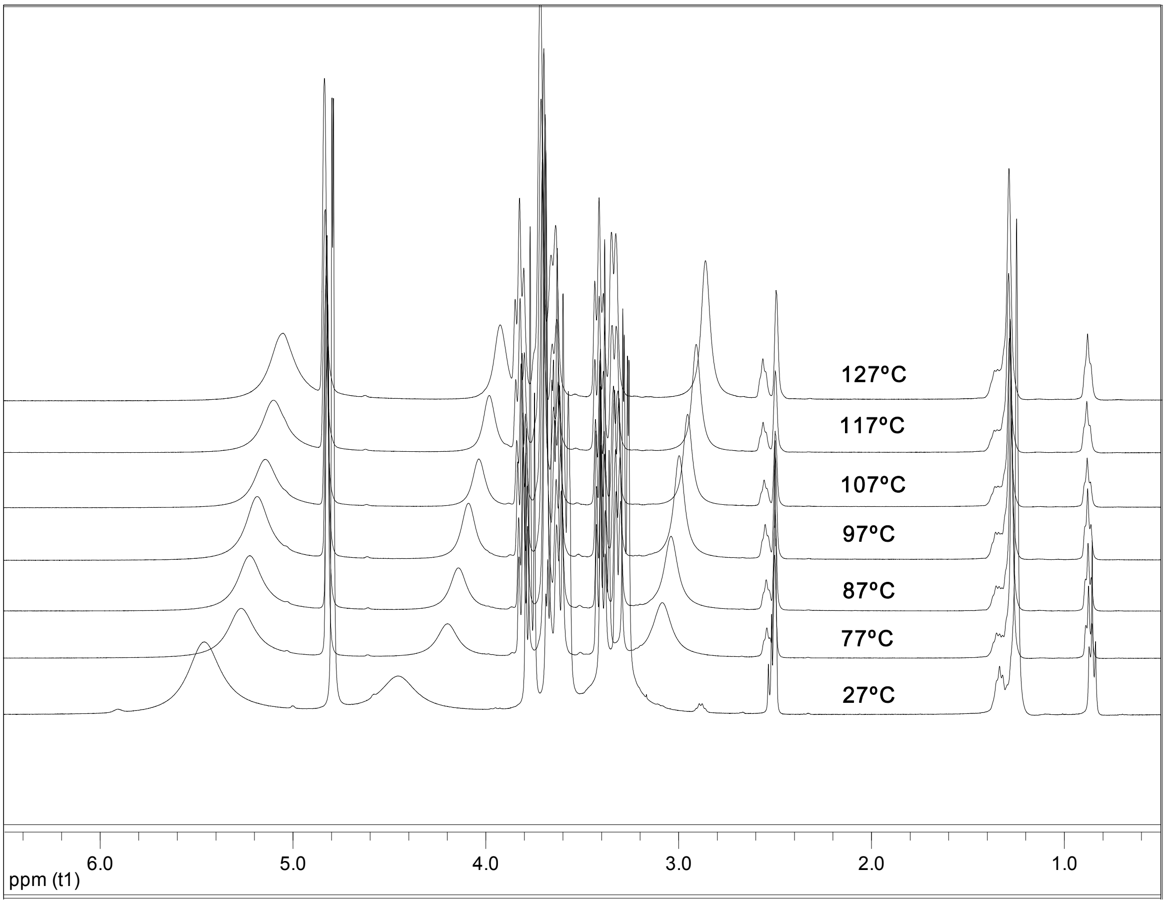

Figure S4. ^{1}H-NMR spectra of an α-cyclodextrin/octylamine IC at various temperatures.

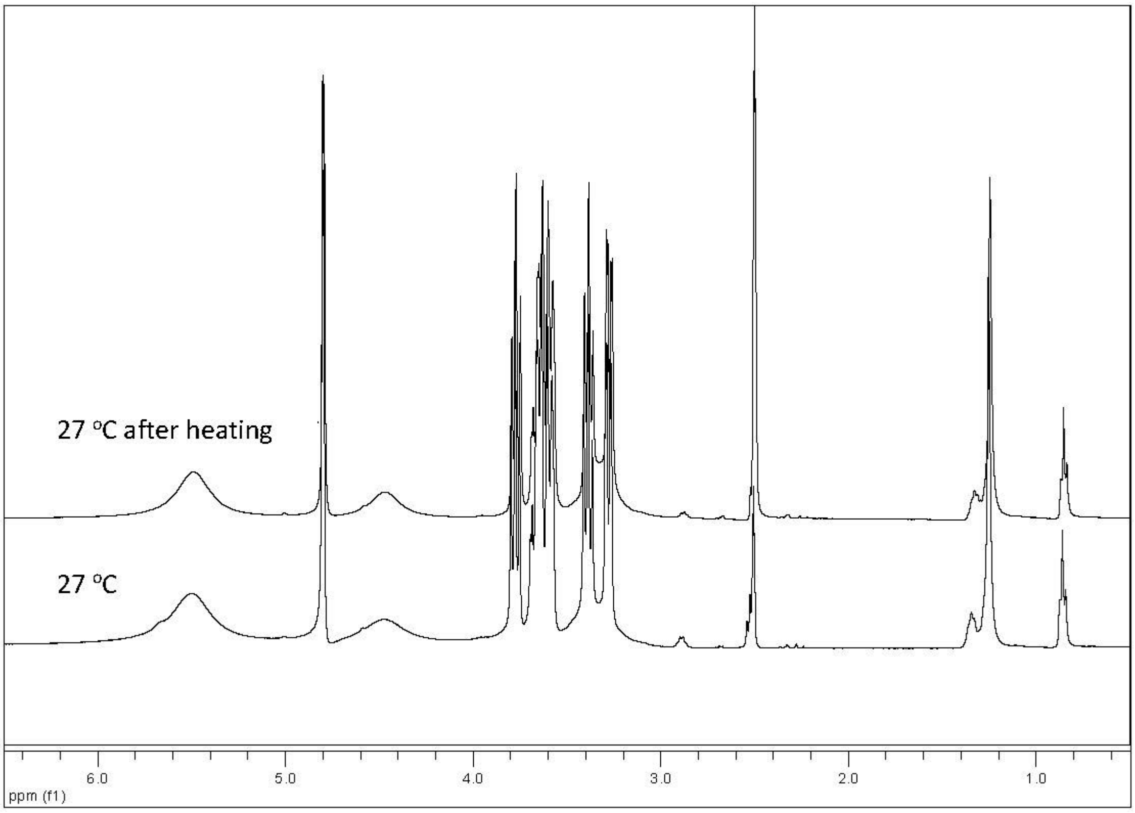

Figure S5. ^{1}H-NMR spectra of the inclusion compound conjugated to AuNPs at 27 °C before and after heating.

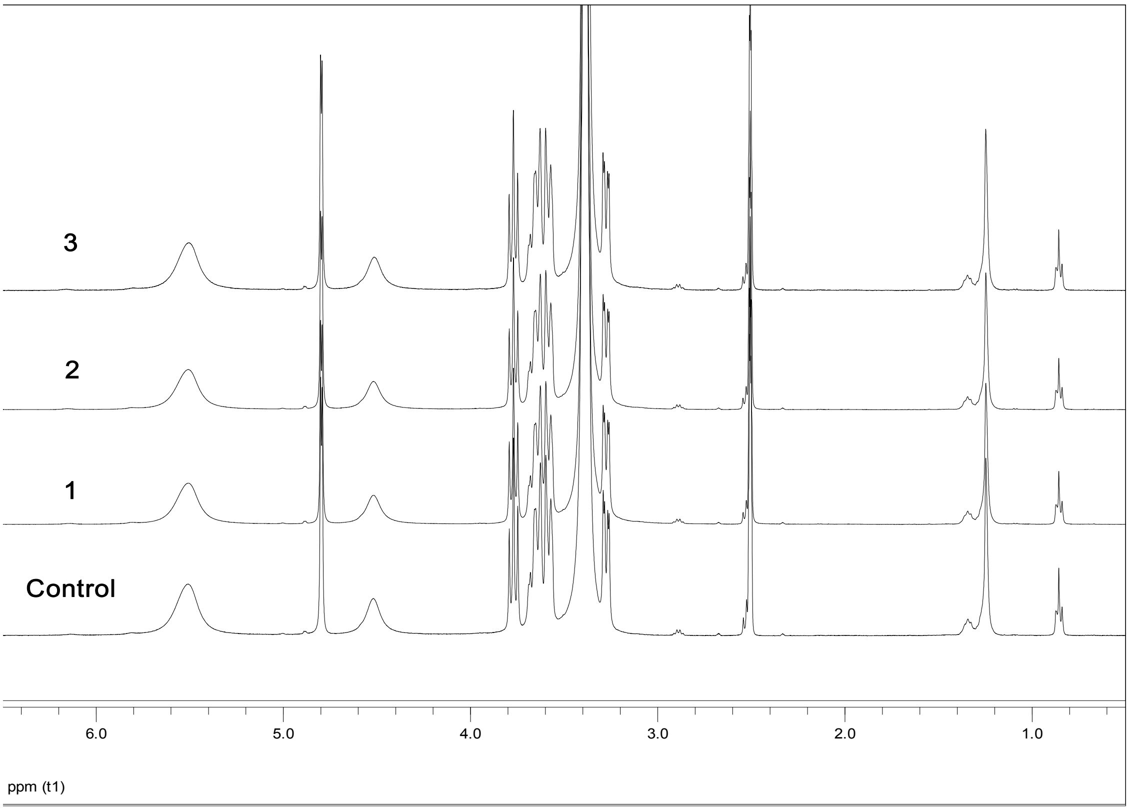

Figure S6. ^{1}H-NMR spectra of IC before and after irradiation. The irradiation conditions for each spectrum were: control (without irradiation), 1 (250 mW/15 min), 2 (450 mW/15 min) and 3 (450 mW/60 min).

Table S1. Chemical shifts of α-CD in the IC at 27 ° C before and after being heated.

Temperature (°C)	H1 (ppm)	H2 (ppm)	H3 (ppm)	H4 (ppm)	H5 (ppm)	H6 (ppm)	OH(2) (ppm)	OH(3) (ppm)	OH(6) (ppm)
27	4.79	3.27	3.77	3.38	3.59	3.65	5.49	5.49	4.47
27 after heating	4.79	3.27	3.77	3.38	3.59	3.65	5.49	5.49	4.47

Table S2. Chemical shifts of OA in the IC to 27 °C before and after being heated.

Temperature (°C)	CH_3 (ppm)	-$(CH_2)_n$- (ppm)	-CH_2- (ppm)	NH_2 (ppm)
27	0.85	1.23	1.31	2.53
27 after heating	0.85	1.23	1.31	2.53
